# Biological effects of olive oil phenolic compounds on mitochondria

**DOI:** 10.1080/23723556.2022.2044263

**Published:** 2022-03-20

**Authors:** Mercedes Blanco-Benítez, Ana Calderón-Fernández, Saray Canales-Cortés, Eva Alegre-Cortés, Elisabet Uribe-Carretero, Marta Paredes-Barquero, Alberto Gimenez-Bejarano, Gema Duque González, Patricia Gómez-Suaga, Juan Ortega-Vidal, Sofía Salido, Joaquín Altarejos, Guadalupe Martínez-Chacón, Mireia Niso-Santano, José M. Fuentes, Rosa A. González-Polo, Sokhna M. S. Yakhine-Diop

**Affiliations:** a Departamento de Bioquímica y Biología Molecular y Genética, Facultad de Enfermería y Terapia Ocupacional, Universidad de Extremadura, Cáceres, Spain; b Instituto de Investigación Biosanitaria de Extremadura (INUBE), Cáceres, Spain; c Centro de Investigación Biomédica en Red de Enfermedades (CIBERNED), Madrid, Spain; d Departamento de Química Inorgánica y Orgánica, Facultad de Ciencias Experimentales, Universidad de Jaén, Jaén, Spain

**Keywords:** Antioxidants, cell death, hydroxytyrosol, Mediterranean diet, mitophagy, oleuropein

## Abstract

Phenolic compounds derived from olive oil have beneficial health properties against cancer, neurodegenerative, and metabolic diseases. Therefore, there are discrepancies in their impact on mitochondrial function that result in changes in oxidative capacity, mitochondrial respiration, and energetic demands. This review focuses on the versatile role of oleuropein, a potent antioxidant that regulates the AMPK/SIRT1/mTOR pathway to modulate autophagy/mitophagy and maintain metabolic homeostasis.

## The Mediterranean Diet

The Mediterranean Diet (MedD) is considered one of the dietary patterns with the greatest accumulated scientific evidence regarding its benefits in human health. In fact, in recent decades, the number of publications on the subject has grown exponentially. The MedD is known as the traditional eating pattern of the countries of the Mediterranean area in the mid-twentieth century. It is characterized by a high consumption of vegetables, legumes, fruit, nuts, whole grains, garlic, onion and spices, a moderate consumption of fish and poultry, the use of virgin olive oil (both for cooking and dressing), the ingestion of wine (consumed in moderation with meals) and a low intake of saturated fat (including that of dairy products), meats, processed meat products and sweets.^1^ The interest of the scientific community on this diet has focused on its role for the prevention or treatment of various associated pathologies with chronic inflammation, such as metabolic syndrome (MS), diabetes, cardiovascular disease (CVD), neurodegenerative diseases and cancer, among others.^2^

The main fatty acid in the MedD are monounsaturated fatty acids (MUFAs), whose intake is related to the reduction of total cholesterol and the increase in high-density lipoproteins (HDL).

The intervention study with the greatest impact regarding the effects of MedD on human health has been the PREDIMED (Prevention with Mediterranean Diet) project, whose main objective was to evaluate the long-term effects of MedD on the incidence of CVD. This multicenter study was carried out in Spain between 2003 and 2011 and included 7,447 men and women older than 50 years with high cardiovascular risk, but with no history of previous cardiovascular events. The participants were randomized into three groups: MedD supplemented with olive oil (1 liter/week), MedD supplemented with nuts (30 g/day) or low-fat diet (control group), without caloric restriction or promotion of physical activity.^3^ After 4.8 years of follow-up, the participants who consumed MedD (supplemented with olive oil or nuts) presented a 30% reduction in the risk of cardiovascular events, mainly cerebrovascular accidents, compared to the control group, with a protective effect of a comparable magnitude to that granted by pharmacological approach such as the use of statins. Additional analyses from the same study reported that the incidence of diabetes was significantly lower among non-diabetic subjects in the MedD groups, with a 52% reduction in the appearance of new cases of diabetes compared to the control group.^4^ The Dietary Guidelines for Americans recommend a “Healthy Mediterranean-Style Eating Pattern” to promote health and protect against common western diseases.^5^

## Characteristic and composition of olive oil

Olive oil has been consumed throughout history from Palestine, where the first crops are located, to South America, passing through the Mediterranean basin. It is obtained from the fruit of the olive tree (*Olea europaea* L.), the olive, a type of drupe characterized by its high oil content, mostly located in its medium part or pulp.

Throughout history, olive oil has been used in different ways, such as mixing it with essences for topical application on the performing massages, for the treatment of skin infections and burns, gastrointestinal disorders and as a fundamental pillar in the culinary culture of the countries that bathe the Mediterranean Sea. All these applications reveal the multiple properties of this oil, being remarkable its properties as a pharmacological agent.^2^

**Figure 1.**
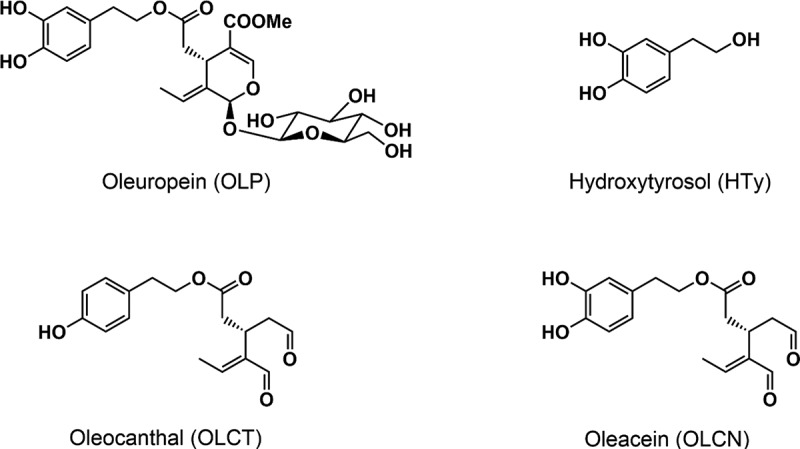
Chemical structures of some phenolic compounds in EVOO. Oleocanthal, oleacein and oleuropein are the main phenolic compounds found in olive oil and fruit. Hydroxytyrosol is the metabolite of oleuropein.

There are two fractions in the composition of this oil. A majority fraction, comprising 97–98% of its total, called the saponifiable fraction, and a minority or non-saponifiable fraction in which various components are found, constituting approximately 2% of the total oil. The majority fraction is essentially composed of triacylglycerides and, in turn, the main fatty acid is oleic acid, a MUFA, although we can find others to a lesser extent, such as linoleic acid, a polyunsaturated fatty acid (PUFA). The minority fraction is made up of more than 200 compounds, among which are the phenolic compounds, such as phenolic acids, phenolic alcohols, lignans, flavonoids and secoiridoids, and secoiridoid-related phenolics, where we find oleuropein (OLP), hydroxytyrosol (HTy), oleocanthal (OLCT), and oleacein (OLCN) (Figure 1), among many others. These compounds are some of the most representative components of the phenolic fraction of olive oil. OLP is found in certain amounts in the fruit (green and ripe olives) and is partially converted into OLCN by endogenous enzymes during the mechanical extraction of the oil and, in turn, OLCN is transformed into HTy. The olive oil phenols give it a unique stability and flavor, and endows this edible oil with greater durability and oxidative stability. The phenolic compounds present here are responsible for the bitter sensation and acidic that produces the oil in the mouth, without forgetting the spicy sensation provided by OLCT and OLCN. Indeed, Andrewes et al. identified, using chromatography techniques, the phenolic compound responsible for the burning and itching sensation of the throat when ingest some virgin olive oils. This compound was originally called “deacetoxyligstroside aglycon,”^2,6^ until Beauchamp et al. in 2005 named it “oleocanthal” (OLCT).^7^

These substances have been related to the healthy properties of olive oil. It is essential to control the growing and harvesting processes of olives to ensure the best and highest quantity of these compounds in the oil, since their concentration will be determined by the maturation stage of the harvest, geographical origin of the olive, irrigation or even the conditions applied during the crushing of the fruit.^8^ There are different qualities of olive oil, being the extra virgin olive oil (EVOO) the richest in minor and predominant compounds in the MedD.

## Phenolic compounds

Phenolic compounds are organic structures that contain at least one hydroxyl group attached to a benzene ring. The form in which they are usually found in nature is linked to a sugar moiety forming glycosides.^9,10^ The Mediterranean area is characterized by being a territory very exposed to sunlight, so many characteristic fruits of the diet of the area, such as grapes or olives, have developed a series of substances that protect against oxidative damage associated with the stress produced by exposure prolonged to ultraviolet light, such as phenols. Therefore, the oils that contain a high proportion of them will be more resistant to degradation. This part of the development and evolution of the fruit becomes important when observing that the incidence of certain pathologies is lower in this region, as we explained previously.^11^

Various phenolic compounds are part of this minor fraction of olive oil whose concentration in it varies depending on various factors such as processing and ripened fruit. However, it is relevant to take into account the proportion of each compound in order to evaluate how its components are absorbed and metabolized, as well as the effects that they have on human health.^12,13^ At least 36 phenolic compounds of EVOO have been identified whose concentration varies according to the type of oil, and whose anti-inflammatory and antioxidant properties make these compounds complement each other.^14^

OLP is a glucoside of an ester of elenolic acid and HTy, with an oleosidic skeleton that is common to the secoiridoid glycosides of plants of the *Oleaceae* family.^15^ OLP and HTy, are loaded with health benefits.^16^ Among the beneficial effects of OLP may be highlighted its antioxidant, anti-inflammatory and antitumor properties, being associated with proliferation and apoptosis by modulating the expression of many signaling pathways, such as the induction of autophagy.^17^

## Oleuropein induces autophagy

Interesting studies have demonstrated that OLP is an autophagy inducer.^18–20^ Autophagy is an important cellular mechanism involved in the clearance of proteins, cytotoxic aggregates, and damaged organelles into lysosomes. It has a key role in cell homeostasis, nutrient deprivation and consequently prevents pathogenesis related to its dysregulation.^21^ Indeed, diseases such as cancer^22^ and neurodegenerative disorders have been associated with autophagy impairment. Generally, autophagy is downregulated in neurodegenerative diseases; however, an upregulation has been reported in certain cases.^23^ According to cancer, the role of autophagy is more complex; it can promote proliferation or suppresses cell transformation.^22^ Consequently, its pharmacological modulation becomes a critical therapeutic strategy to control those diseases. In this line, several autophagy modulators have emerged and scientists are still interested in finding new ones from our daily diet, and this is the case with OLP. It exists different types of autophagy depending on the manner by which the cargo is cleared: microautophagy, chaperone-mediated autophagy, and macroautophagy.^24^ All of them need functional acidic lysosomes to complete the degradation and the recycling of products.

Chaperone-mediated autophagy involves the Heat Shock Cognate 70 (Hsc70) chaperone in the selection of the KFERQ motif in proteins that will translocate into lysosomes.^21,24^ Microautophagy is a nonselective degradation that is characterized by a sequestration of cytosolic components through an invagination of the lysosomal membrane.^24^ Finally, macroautophagy, often called autophagy, collects the cytosolic cargoes into some double-membrane vesicles namely autophagosomes that will later fusion with lysosomes to constitute lately the autophagolysosomes/autolysosomes.^24^ Such a process can be selective, when it promotes the sequestration and degradation of specific contents leading to a sub-classification of macroautophagy: mitophagy for mitochondria, reticulophagy for endoplasmic reticulum (ER), ribophagy for ribosomes, etc.

OLP is able to modulate autophagy in various cell types (Table 1).

**Table 1.** Oleuropein induces autophagy in different cell lines

Cell lines	OLP treatments	Observations	References
Cardiomyocytes	100 µM	↑BECN1, ↑LC3-II	^19^
SH-SY5Y cells	50 µM	↑BECN1	^20^
N2a cells	9–100 µM	↑BECN1,↑ p62, ↑LC3-II	^18^
RIN-5F cells	50 µM	↑LC3-II	^20^

**Figure 2.**
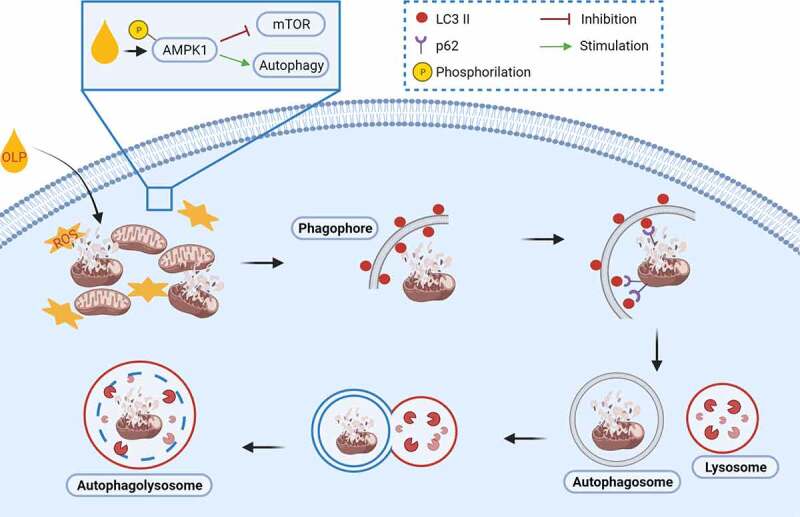
Oleuropein-Induced autophagy. Oleuropein induces autophagy by activating AMPK (phosphor-AMPK Thr172) and inhibiting mTOR. Administration of oleuropein improves autophagosome formation and lysosome acidification, which promotes decreased generation of reactive oxygen species (ROS) through the clearance of damaged mitochondria (called mitophagy).

However, it may act in multiple pathways to accomplish the autophagy-induced beneficial effects against cancer, neurodegenerative,^18^ cardiovascular,^19^ and metabolic diseases.^25^ It is noteworthy to highlight that the incubation time and the used concentrations have a great impact on OLP-mediated response. For a short time, treatment in SH-SY5Y neuroblastoma cells, autophagy induction by 50 µM OLP results in an intermittent release of calcium (Ca^2+^) from the ER to activate the Ca^2+^/calmodulin-dependent protein kinase kinase-beta (CAMMKβ) which then phosphorylates AMP-activated protein kinase (AMPK).^20^ The same concentration of OLP used in N2a cells, for 3–48 h, decreases the phosphorylation of the mammalian target of rapamycin (mTOR) substrate, p70S6K. Moreover, this inhibition of the mTOR pathway was more efficient with 50–100 µM OLP for 6 h.^18^ The implication of mTOR in OLP-induced autopahgy is corroborated in resting cardiomyocytes through the nuclear translocation of the transcription factor EB (TFEB) and the upregulation of its target genes SQSTM1/p62, lysosomal associated membrane protein 1(LAMP1) and ATP6V1.^19^ TFEB is the master regulator of lysosomal biogenesis. Indeed, when activated, mTOR phosphorylates TFEB, the latter is sequestered in the cytoplasm. The inhibition of mTOR and/or the calcineurin phosphatase activate TFEB and facilitate its translocation into the nucleus, where it exerts its activity. In contrast, high concentrations (200 µM and 400 µM) of OLP activated the AMPK pathway, and not the phosphatidylinositol-3 kinase (PI3K)/AKT pathway^16^ in C2C12 muscle cells. Independently of the cell lines used in cell culture, OLP increases BECN1,^18–20^ SQSTM1^18^ and LC3-II^18–20^ proteins. Unexpectedly, picomolar dose of OLP inhibits autophagosome formation and decreases lysosome acidification in neuronal PC12 cells.^26^ All these data suggest that OLP might modulate mitochondrial function and mitochondrial energy, and, as an autophagic inducer, it might also promote the elimination of damaged mitochondria (Figure 2).

## Oleuropein and mitochondria

Mitochondria are the major source of energetic cell production, but also the main producer of ROS generation.^27^ Moderating ROS production is indispensable for the regulation of different signaling pathways. However, mitochondrial dysfunction and uncontrolled production of ROS^28^ underlying oxidative stress are the common hallmarks of multiple diseases. Therefore, there is a very good reason to believe that the balance depends on the regulation or the reestablishment of antioxidant levels. For instance, a chronic OLP (60 mg/kg/day for 8 weeks) administration decreases the oxidative stress levels in spontaneously hypertensive rats, by upregulating the antioxidant activities of (SOD) and glutathione (GSH).^29^ Similar results were observed with HTy in MC3T3-E1 cells.^30^ OLP and/or HTy increase the expression of Nrf2, its nuclear translocation and the Nrf2-induced antioxidant enzymes NADPH Quinone Dehydrogenase 1 (NQO1) and heme oxygenase-1 (HO-1).^29–31^ In addition to their ability to scavenge the mitochondria-generated ROS, OLP and HTy foster the *novo* synthesis of mitochondria as evidenced by the expression of the mitochondrial transcription factor a (TFAM), the increase in mitochondrial DNA (mtDNA) copy number and mitochondondrial mass.^32,33^ Consistent with these effects, 50 µM OLP increases the SIRT1 protein in avian muscles cells, which activates peroxisome proliferator-activated receptor-gamma (PPARγ) coactivator (PGC1-α) through its deacetylase activity.^32^ PGC1-α is associated with mitochondrial biogenesis. Its upregulation improves mitochondrial function, resulting in an increasing COX activity.^32^ In fact, the knockdown of PGC1-α gene abolished the HTy-improved mitochondrial activity.^33^ While OLP enhances the mitochondrial complexes II and IV proteins,^32^ its metabolite can significantly increase the expression of all mitochondrial complexes, with some discrepancies in *in vivo*^31^and *in vitro*.^33^ SIRT1 is a histone deacetylase (HDAC) class III critical for cell survival; its inhibition provokes mitochondrial fragmentation.^34^ Besides its role in mitochondrial biogenesis, SIRT1 induces autophagy and mitophagy in HTy-treated primary chondrocytes.^35^ Gene silencing of SIRT1 impedes the HTy-mediated autophagosome formation^35^ and probably leading to mitochondrial accumulation. There are not enough studies on the direct role of OLP and HTy in the regulation of mitophagy. But all activated signaling pathways allow us to state that both have the potential to eliminate dysfunctional mitochondria, as they activate AMPK^33,36^ and the latter promotes PINK1 phosphorylation (Ser495) that retrieves PARKIN to mitochondria.^37^ In addition, HTy upregulates gene expression of PTEN-induced kinase 1 (*PINK1*) in oleic acid-treated hepatocytes.^23^ OLP induces mitochondrial fission over fusion by increasing dynamin related-protein (DRP1), while MFN2 was decreased.^29^ In this study, Sun et al. consider that the recovering of mitochondrial proteins is due to an attenuation of mitochondrial degradation despite the OLP-induced DRP1.^29^ Indeed, these finding were corroborated by another report, where a blockade of excessive mitochondrial fragmentation through the inhibition of DRP1 activity by OLP was described.^38^

In contrast to above mentioned, OLP has been reported to provoke a mitochondrial membrane potential loss in breast and ovarian cancer cells, and to significantly enhance the mitochondrial ROS production.^39^ OLP (150 µM) exerts a selective toxicity via the inhibition of AKT pathway, in non-small-cell lung cancer (NSCLC), mediated by the increase of SOD2.^40^ OLP affects cell proliferation in several cancer cell models such as breast, lung, pancreas, ovarian by inhibiting the AKT pathway and activating the intrinsic apoptotic pathway in two different mechanisms either reducing or increasing ROS levels. So, despite the decrease of superoxide anion, OLP elicits apoptosis through an interaction between the mitochondrial glyoxalase 2 and Bax proteins.^40^

## Protective effect of oleuropein

The cytoprotective effect of OLP and its metabolite is associated with the reduction of proteins aggregates toxicity,^41^ the attenuation of oxidative stress^26^ and even pro-oxidant,^39^ the improvement of neuronal function,^42^ and the autophagy-induced cell death in cancer cells. All these effects highlight the versatile role of OLP, its potential to protect in different ways and under varying cellular conditions. HTy reduce H_2_O_2_-induced DNA damage and cell death. However, while the cytoprotective effect of HTy is related to autophagy, the nucleic acid protection is due to its antioxidant effect.^35^ Meanwhile, OLP protects against tyramine-induced oxidative stress in cardiomyocytes by restoring autophagy flux through TFEB activation.^19^ In fact, the overactivation of monoamineoxidase A inhibits autophagy in tyramine-treated cardiomyocytes, which trigger an accumulation of dysfunctional mitochondria, increasing ROS generation and necrosis. In TgCRND8 mice (Alzheimer disease model)^20^ and rotenone-treated *Caenorhabditis elegans*,^43^ OLP improves movement behavior and decreases the amyloid plaques via the inhibition of mTOR and the activation of AMPK.^20^ In the case of Ischemia/reperfusion injury (IRI), OLP exerts its neuroprotective effect by increasing cognitive function, neurotrophic factors and protecting against apoptotic neuronal death through the activation of AKT pathway.^44^ However, other studies attribute the neuroprotective effect of OLP, in 6-OHDA-induced apoptosis in neuronal PC12 cells,^45^ to its anti-oxidative property followed by a decrease in the level of the pro-apoptotic BAX protein^25^ excluding the role of autophagy.

## Metabolic changes

OLP is considered a caloric-restriction mimetic because of its ability to modulate the AMPKα/SIRT1/mTOR pathway. AMPKα and SIRT1 positively regulate autophagy, in contrary to AKT/mTOR pathway.^46^ Beyond autophagy, the activation of AMPK (phosphor-AMPKα Thr172) inhibits via phosphorylation the Acetyl-CoA carboxylase (ACC, phosphor-ACC Ser79), which triggers the β-oxidation of fatty acid in mitochondria.^29^ Accordingly, hepatic mitochondrial dysfunction leads to abnormal lipid metabolism. Likewise, a high-fat diet is prone to induce damaged mitochondria, driving a lower ATP content, an increase of triacyglycerols and cholesterol.^26,40^ HTy reduces the gene expression of *fatty Acid Synthase* (*FASN*) and increases those of *PPARα, carnitine palmitoyltransferase −1* (*CPT1*),^33^ that is likely to induce fatty acid oxidation and to attenuate lipid accumulation in Fish (*M. amblycephala*) fed high-fat diet^26^ as well as in 3T3-L1 adipocytes and enhance the oxygen consumption rate.^33^ On the other hand, OLP activated-AMPKα/ACC signaling increases glucose uptake in C2C12 cells. Most importantly, OLP does not hinder the regulatory function of insulin regarding glucose metabolism in these myotubes, and further boosts the redistribution of the glucose transporter-4 (GLUT4) to cell membrane.^16^ However, in BRAF melanoma cells, 200 µM OLP inhibits the glycolytic metabolism of primary and metastatic melanoma cells, downregulating the expression of GLUT1, pyruvate kinase 2 and monocarboxylate transporter 4 (MCT4).^47^ The beneficial effects of OLP on glucose and lipid metabolism sustain mitochondrial respiration and cellular energy demand in order to cope with induced or generated oxidative stress. OLP and/or HTy improve metabolic homeostasis through the mitochondrial function that is intimately regulated by the induction of mitochondrial biogenesis and mitophagy.

## Conclusions

There is currently no clear evidence of the efficacy of these olive oil phenolic compounds. Not because they do not have healthy properties, but for the reason that there are no conclusive data. It is very difficult to demonstrate and find a single nutrient that acts specifically in many diseases, including cancer and neurodegenerative diseases, that develop over several years and where multiple variables such as lifestyle habits (physical activity, smoking …) and genetic predispositions have a great influence.

## Abbreviations

ANSI: American National Standards Institute
CMS: Centers for Medicare and Medicaid Services
HCPCS: Healthcare Common Procedure Coding System
ISO: International Organization for Standardization
RESNA: Rehabilitation Engineering and Assistive Technology Society of North America
VGM: Van G. Miller Group
WMS: wheeled mobility and seating
WRR: Wheelchair Repair Registry
